# Diagnostic potential of serum exosomal colorectal neoplasia differentially expressed long non-coding RNA (CRNDE-p) and microRNA-217 expression in colorectal carcinoma

**DOI:** 10.18632/oncotarget.19407

**Published:** 2017-07-20

**Authors:** Bo Yu, Qiong Du, Huan Li, Hong-Yue Liu, Xuan Ye, Bin Zhu, Qing Zhai, Xin-Xiang Li

**Affiliations:** ^1^ Department of Oncology, Shanghai Medical College, Fudan University, Shanghai 200032, China; ^2^ Department of Pharmacy, Fudan University Shanghai Cancer Center, Shanghai 200032, China; ^3^ Department of Colorectal Surgery, Fudan University Shanghai Cancer Center, Shanghai 200032, China

**Keywords:** colorectal carcinoma, exosome, CRNDE-p, miR-217

## Abstract

In this study, we investigated the diagnostic potential of serum exosomal colorectal neoplasia differentially expressed (CRNDE-p) long coding RNA and microRNA-217 in colorectal carcinoma (CRC). We detected high CRNDE-p and low miR-217 levels in exosomes released by multiple CRC cell lines into culture media as well as in sera from CRC xenograft mice and CRC patients. Conversely, we observed lower CRNDE-p and higher miR-217 levels in serum exosomes from post-chemotherapy than from pre-chemotherapy patient samples. The area under curve (AUC) value for the serum exosomal CRNDE-p and miR-217 combination was higher than CRNDE-p or miR-217 alone. Moreover, high CRNDE-p and low miR-217 serum exosomal levels correlated with advanced clinical stages (III/IV), tumor classification (T3/T4), and lymph node or distant metastasis. Thus combined evaluation of serum exosomal CRNDE-p and miR-217 levels show diagnostic and prognostic potential for CRC patients.

## INTRODUCTION

Colorectal carcinoma (CRC) is one of the main causes of cancer mortality worldwide, accounting for 15% of all newly diagnosed cancers. The median age of newly diagnosed patients is between 60 and 80 years of age [1–3]. Early diagnosis and treatment significantly improves the overall survival rate of CRC patients [4, 5]. Colonoscopy provides the highest diagnostic accuracy, but is inconvenient and invasive and therefore limited in its use for preliminary diagnosis. Therefore, plasma and serum biomarkers are critical for CRC diagnosis [6–8]. The preferred biomarker is carcinoembryonic antigen (CEA), which exhibits low sensitivity and specificity particularly in the early stages of CRC. Hence, new CRC-specific biomarkers are urgently required to complement and improve the current CRC diagnostic strategies.

Interaction of tumor cells with their microenvironment is a critical determinant of the carcinogenesis and its progression including invasiveness and metastasis potential [9]. Microvesicles (MVs) are important intercellular carriers of various signaling molecules that play critical roles in tumor metastasis [10, 11]. Exosomes are small cell-specific double membrane microvesicles with a diameter of approximate 40–100 nm [12]. They are distributed in body fluids such as peripheral blood, urine, saliva, ascites and amniotic fluid [13]. Many different cell types release exosomes that carry secretory proteins, lipids and nucleic acids including tumor-derived noncoding RNA (ncRNAs) [14].

Aberrant expression of long noncoding RNAs (lncRNAs), which are ≥ 200 nucleotides long with limited protein-coding potential is involved in the tumor progression [15, 16]. LncRNAs are potential tumor biomarkers due to their pivotal role in tumor development or suppression [17, 18]. The Colorectal Neoplasia Differentially Expressed (*CRNDE*) gene was first identified as a potential prognostic factor in ovarian cancer patients [19]. Until now, at least 10 splice variants of CRNDE have been identified. Several studies have demonstrated high CRNDE expression in colon cancer tissues than in normal colon mucosa [20, 21]. CRNDE is over-expressed in colorectal carcinomas and promotes *in vitro* and *in vivo* growth and invasion of CRC cells [22]. Therefore, CRNDE is a promising biomarker candidate in colorectal carcinogenesis. However, it is difficult to obtain tissue biopsies from suspected CRC patients. Thus, finding a noninvasive, sensitive, and cost effective nucleic acid tumor marker for CRC diagnosis has remained a major challenge.

Recent studies have demonstrated that RNAs such as mRNAs, microRNAs and lncRNAs are secreted via exosomes from tumor cells into body fluids like blood, urine, milk, and saliva [23]. Circulating exosomes carrying regulatory RNA molecules play a critical role in long distance cell-cell communication [24]. In this study, we detected the transcript 5 of CRNDE (CRNDE-p, NM_001308963.1) and miR-217 in circulating exosomes to investigated if exosomal CRNDE-p and miR-217 levels have diagnostic relevance in CRC patients.

## RESULTS

### Characterization of purified serum exosome fractions

**Figure 1:**
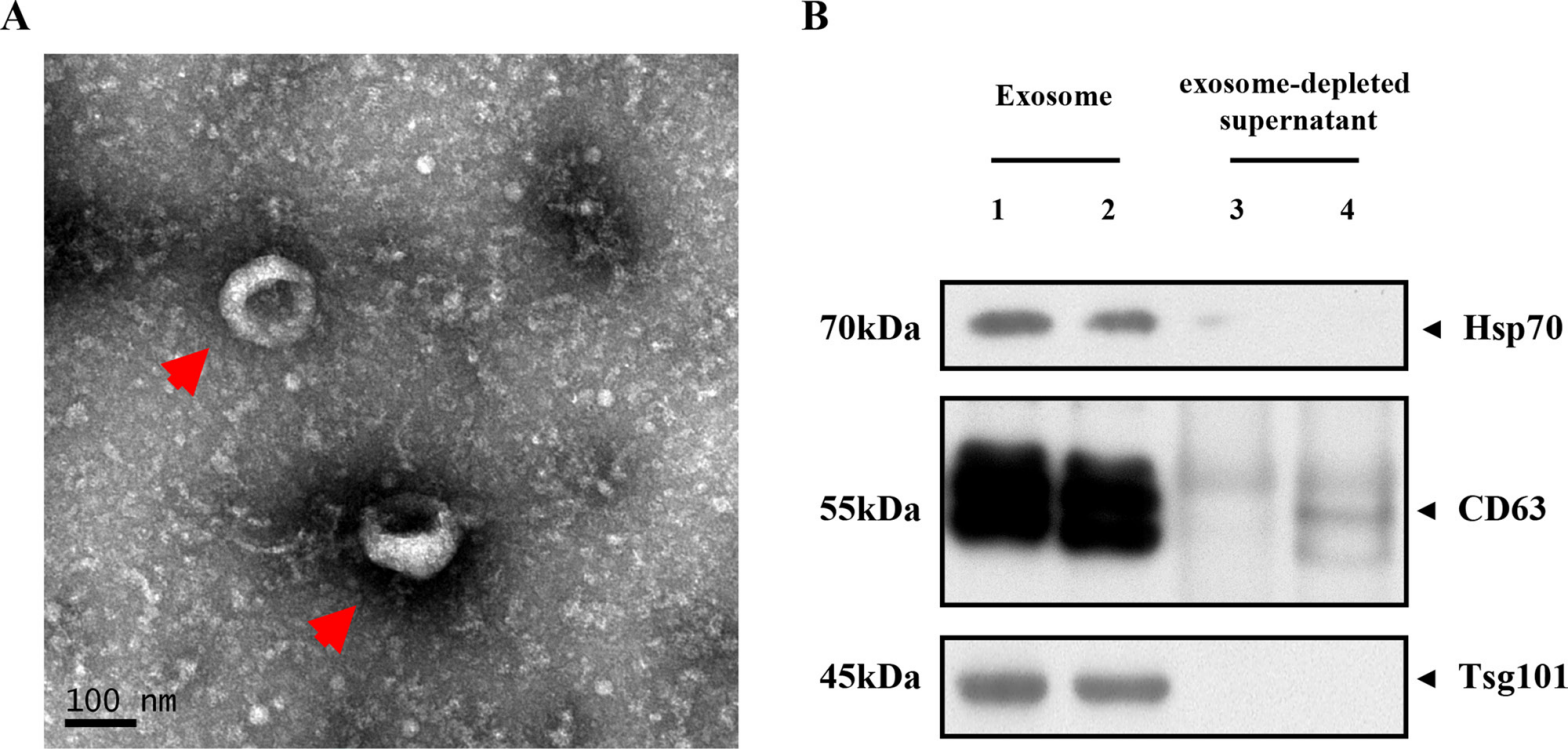
Characterization of serum exosomes (**A**) Representative TEM image shows enriched serum exosomes as indicated by the arrow (scale bar: 100 nm; HV = 80.0 kv); (**B**) Representative western blots showing Hsp-70, CD63 and TSG101 levels in purified serum exosomes from CRC patients (lanes 1–2) and exosome-depleted supernatant (lanes 3–4).

The purified serum exosomes isolated from patient and control subjects were analyzed by transmission electron microscopy. As shown in Figure 1A, the exosomes were round cystic double membrane vesicles, 60 nm to 120 nm in diameter. Western blot analysis demonstrated that the serum exosomal preparations were enriched in specific exosomal marker proteins, namely, CD63, TSG101 and Hsp70 (Figure 1B).

### CRNDE-p and miR-217 levels in exosomes from human CRC cell lines and CRC mouse xenograft model

**Figure 2:**
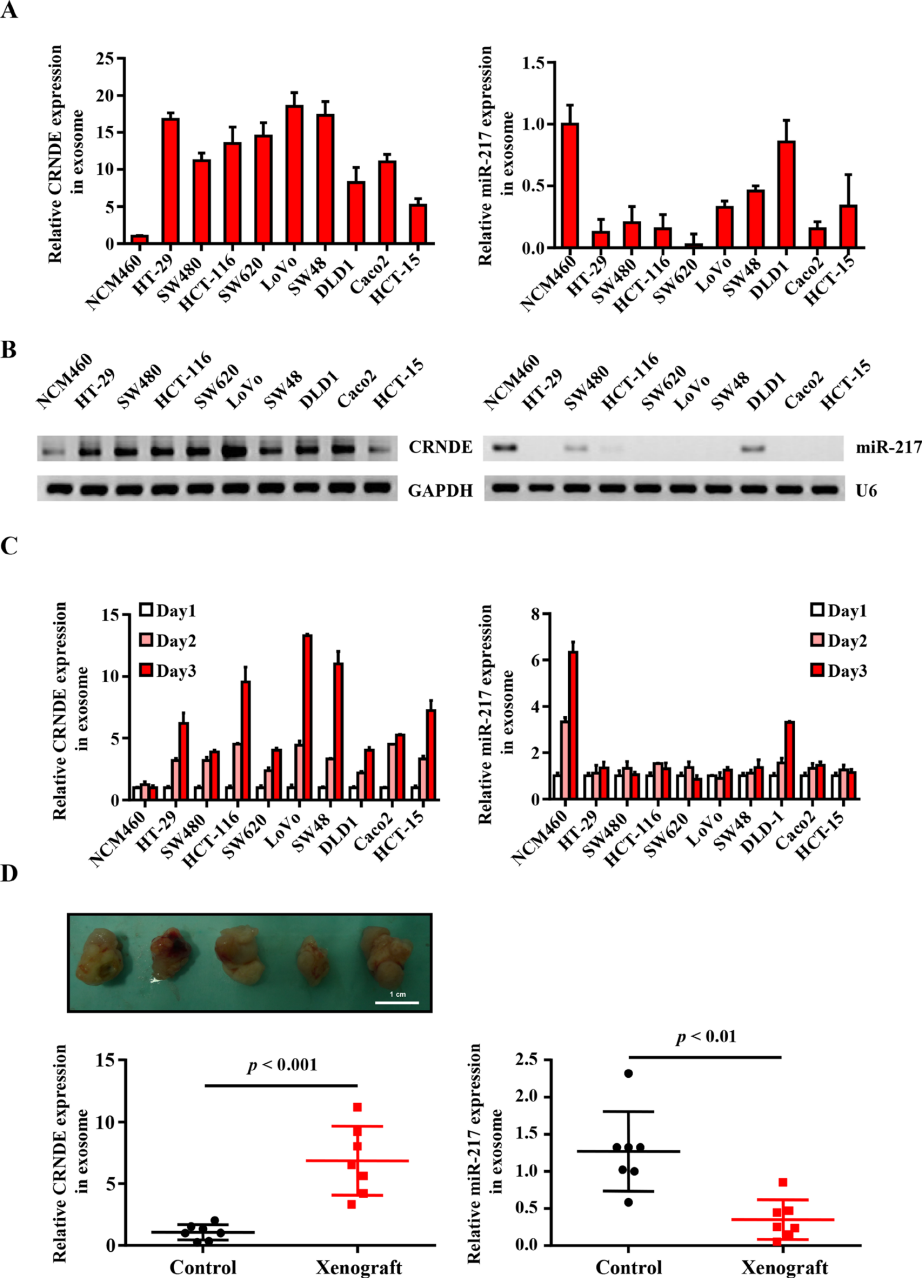
Differential expression of exosomal CRNDE-p and miR-217 from human CRC cell lines and CRC mouse xenograft model (**A**) Relative expression of CRNDE-p and miR-217 in exosomes isolated from the culture supernatants of normal human colon mucosal epithelial cell line NCM460 and human colorectal cancer cell lines, namely, HT-29, SW480, HCT-116, SW620, LoVo, SW48, DLD-1, Caco2 and HT-15 lines. (**B**) Northern Blot is used to evaluate the expression of CRNDE-p and miR-217 in exosomes isolated from the mentioned cells. (**C**) Relative expression of CRNDE-p and miR-217 in exosomes isolated from the culture supernatants on days 1–3 of normal human colon mucosal epithelial cell line NCM460 and human colorectal cancer cell lines, namely, HT-29, SW480, HCT-116, SW620, LoVo, SW48, DLD-1, Caco2 and HT-15 lines. (**D**) Representative image of tumors from xenograft mice at 5 weeks after HCT-116 cells’ injection. Relative expression of serum exosomal CRNDE-p and miR-217 in CRC xenograft mice and sham controls (*n* = 6). Note: The expression of CRNDE-p and miR-217 were quantified relative to cel-miR-39 by qRT-PCR.

Next, we analyzed the expression of CRNDE-p and miR-217 in exosomes from normal human colon mucosal epithelial cell line NCM460 and multiple human colorectal cancer cell lines, namely, HT-29, SW480, HCT-116, SW620, LoVo, SW48, DLD-1, Caco2 and HT-15. We observed high CRNDE-p and low miR-217 levels in exosomes released from CRC cells than in exosomes released from the control NCM460 cells by quantitative real-time-PCR (Figure 2A) and Northern blot (Figure 2B). Moreover, exosomal CRNDE-p expression increased over time in the CRC cell lines, especially in LoVo cells, but was constant in NCM460 cells over time (Figure 2C). Conversely, exosomal miR-217 levels increased over time in the NCM460 cells, but remained constant in most CRC cell lines except DLD-1 cells (Figure 2C). These data suggested an inverse relationship between exosomal CRNDE-p (high) and miR-217 (low) expression in CRC cell lines.

Next, we investigated CRNDE-p and miR-217 levels in exosomes isolated from the serum of sham and CRC mouse xenograft model, 5 weeks after transplantation when the tumors were well established. We observed high CRNDE-p and low miR-217 levels in the serum exosomes from the CRC xenograft mice than in sham controls (Figure 2D). Therefore, our results suggested an inverse correlation between exosomal CRNDE-p and miR-217 in both human CRC cell lines and the CRC mice xenograft model.

### Differential expression of CRNDE-p and miR-217 in serum exosomes from CRC patients, adenoma patients and health volunteers

**Figure 3:**
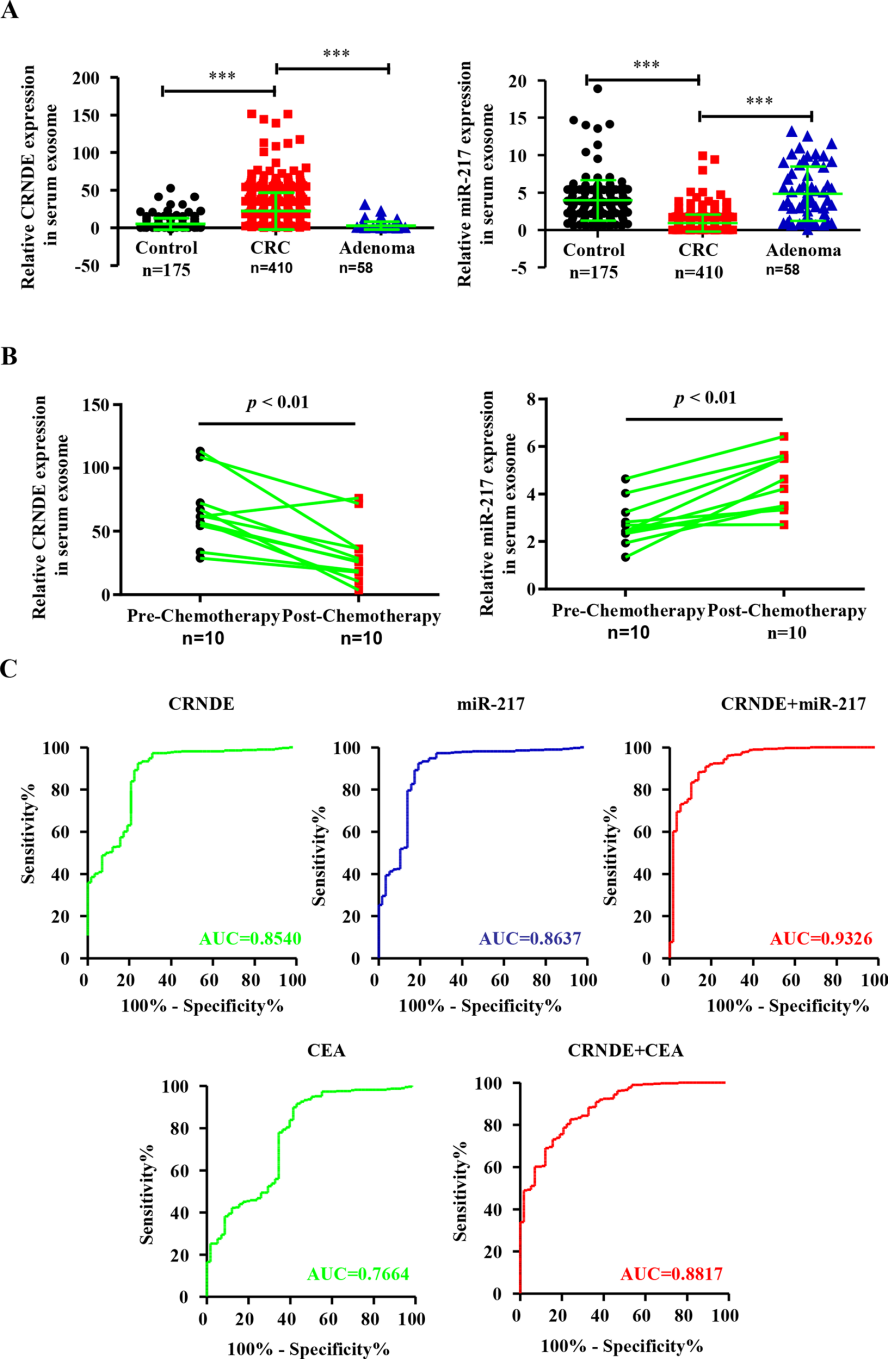
Differential expression of exosomal CRNDE-p and miR-217 in serum exosomes from CRC patients, adenoma patients and health volunteers and ROC curve analysis (**A**) Relative expression of serum exosomal CRNDE-p and miR-217 in CRC patients (*n* = 410), adenoma patients (*n* = 58) and healthy volunteers (*n* = 175). (**B**) Relative levels of serum exosomal CRNDE-p and miR-217 in CRC patients before (Pre-chemotherapy) and 21 days after chemotherapy (Post-chemotherapy). (**C**) Receiver-operating characteristic (ROC) curves of serum exosomal CRNDE-p, miR-217, CEA and combined CRNDE-p/miR-217 or CRNDE-p/CEA levels in CRC patients and adenoma patients.

Next, we analyzed the exosomal CRNDE-p and miR-217 in CRC patients by qRT-PCR by normalizing to cel-miR-39 as an internal control. We observed high CRNDE-p (Figure 3A) and low miR-217 (Figure 3B) levels in the exosomes from the serum of CRC patients than in adenoma patients or healthy subjects. Furthermore, we compared the exosomal CRNDE-p and miR-217 levels in paired pre-chemotherapy and post-chemotherapy 10 patients’ serum samples. We observed low CRNDE-p (Figure 3B) and high miR-217 levels in post-chemotherapy samples than in pre-chemotherapy samples.

### Association between clinicopathological characteristics of CRC patients and serum exosomal CRNDE-p and miR-217 levels

The Receiver operating characteristic (ROC) curve analyses for discriminating CRC patients from adenoma group showed that area under curve (AUC) values for exosomal CRNDE-p and miR-217 were 0.8540 (95% CI: 0.8235–0.9263) and 0.8637 (95% CI: 0.8405–0.9470), respectively (Figure 3C). This suggested that both factors could strongly discriminate CRC patients from healthy individuals. Moreover, the AUC value for both factors in combination by logistic regression was 0.9326 (Figure 3C). Conventional tumor marker carcinoembryogenic antigen (CEA) was also determined and compared diagnostic power with exosomal CRNDE-p. The AUC was 0.7664 (95% CI: 0.6944–0.8383) for CEA alone and 0.8817 (95% CI: 0.8372–0.7262) for CEA/CRNDE-p combination. This suggested that the combined CRNDE-p/miR-217 had better diagnostic potential than either individual factors alone or combined CRNDE-p/CEA. The association between clinicopathological features of CRC patients and the exosomal CRNDE-p and miR-217 levels is shown in Table 1. We observed high CRNDE-p and low miR217 in the serum exosomes correlated with advanced T-stages (T3 and T4), lymph node metastasis and clinical stages (III and IV) as shown in Table 1. However, there was no significant correlation between exosomal CRNDE-p and miR-217 expression with the age, gender and location of CRC patients.

**Table 1:** Correlations of clinicopathological parameters and expression level of CRNDE and miR-217 in patients with CRC (n = 410)

Varibles	No. of patient	No. of patient (%)	CRNDE expression (Mean ± SEM)	*P*	miR-217 expression (Mean ± SEM)	*P*
Ages (years)						
< 59	206	50.24	21.14 ± 3.58	0.6672	3.17 ± 0.22	0.2765
≥ 59	205	50.00	23.58±4.40		2.72 ± 0.35	
Gender						
Male	252	61.46	22.01 ± 3.02	0.8418	2.85 ± 0.31	0.5488
Female	158	38.54	22.93 ± 3.26		3.12 ± 0.28	
Localization						
Rectum	181	44.15	22.74 ± 1.85	0.8131	2.75 ± 0.28	0.4307
Colon	229	55.85	22.07 ± 2.05		3.11 ± 0.34	
T stage						
T1–T2	204	49.76	15.65 ± 1.77	0.0033	3.74 ± 0.42	0.043
T3–T4	206	50.24	26.10 ± 3.05		2.17 ± 0.35	
Regional lymph node metastasis						
Negative	235	57.32	16.52 ± 2.22	0.0007	3.47 ± 0.36	0.011
Positive	175	42.68	30.21 ± 3.59		2.26 ± 0.26	
Distant metastasis						
No	305	74.39	20.77 ± 1.36	0.0293	3.25 ± 0.33	0.0479
Yes	105	25.61	26.99 ± 2.81		2.09 ± 0.27	
Clinical stage						
I–II	200	48.78	18.85 ± 2.95	0.0436	3.58 ± 0.29	0.0038
III–IV	211	51.46	25.59 ± 2.05		2.34 ± 0.31	

## DISCUSSION

Currently, the preoperative diagnostic methods for colorectal carcinoma include colonoscopy, clinical palpation and imaging techniques. Although conventional biopsy from the primary tumor can accurately diagnose CRC, it is limited because it's invasive and requires local or general anesthesia. The non-invasive imaging techniques such as CT, MRI, ultrasonography, and positron emission tomography (PET) are restricted for diagnostic purposes because they are not economical [2, 4]. Therefore, more effective diagnostic tools are necessary because early diagnosis is critical to improve the survival rates of CRC patients.

Diagnostic testing with human blood is a traditional, non-invasive and convenient method [5, 6]. Circulating noncoding RNAs such as miR-21, MALAT-1, HOTAIR have been used as biomarkers for cancer diagnosis [25, 26]. In this study, we analyzed serum exosomal CRNDE-p and miR-217 levels in subgroups of CRC patients to identify association with clinicopathological parameters such as clinical staging, tumor classification, and lymph node and distant metastasis. We observed high CRNDE-p and low miR-217 in the serum exosomes from patients with tumor stage T3/T4, clinical stage III/IV, lymph node and distant metastasis than in patients with tumor stageT1/T2, clinical stage I/II and without lymph node and distant metastasis. This demonstrated an inverse relationship between serum exosomal CRNDE-p and miR-217 in association with CRC. Since, miR-217 does not bind CRNDE-p in the exosomes, we hypothesized that CRNDE-p probably binds to miR-217 only in the intracellular environment. Further investigations are required to unravel the underlying mechanism of low exosomal miR-217 levels. In general, the CRNDE-p expression was higher and miR-217 expression was lower in the serum exosomes isolated from CRC patients than in healthy subjects. This suggested that either or both these serum ncRNAs were potential diagnostic biomarkers for CRC.

To confirm the diagnostic value of serum exosomal CRNDE-p and miR-217 in CRC, we performed ROC curve analysis and determined their cutoff values in CRC patients and health subjects. The area under curve (AUC) values demonstrated that expression of both exosomal CRNDE-p and miR-217 demonstrated high sensitivity and specificity and clearly, distinguished CRC patients from benign diseases. Moreover, the AUC value for combined expression of serum exosomal CRNDE-p and miR-217 was markedly higher than either CRNDE-p, miR-217, CEA alone or combined CRNDE-p/CEA. This suggested that the combination of CRNDE-p and miR-217 was a better diagnostic predictor than any applicated conventional tumor marker CEA.

Another interesting finding in this study was that the CRNDE-p levels were aberrantly high in pre-chemotherapy serum samples, but lower in post-chemotherapy serum samples. Conversely, miR-217 levels were aberrantly low in pre-chemotherapy serum samples, but high in post-chemotherapy samples. This suggests that serum exosomal CRNDE-p and miR-217 levels were potential prognostic biomarkers for CRC patients.

In conclusion, our study demonstrates that serum exosomal CRNDE-p and miR-217 levels in combination show diagnostic and prognostic potential for CRC.

## MATERIALS AND METHODS

### Patients and serum samples

We enrolled 411 CRC patients, 58 adenoma patients and 175 healthy volunteers in this study, which was approved by the Ethics Committee of Fudan University Shanghai Cancer Center. TNM classification was based on the tumor pathology after surgery. Peripheral blood samples were collected in EDTA-tubes from the study subjects and centrifuged at 3,000 × g for 15 min at 4°C. Then, 500 μl of supernatants were aliquoted into eppendorf tubes and stored at –80°C for further use.

### Cell lines

The normal human colon mucosal epithelial cell line, NCM460 and human colorectal cancer cell lines (HT-29, SW480, HCT-116, SW620, LoVo, SW48, DLD-1, Caco2 and HT-15) were purchased from American Type Culture Collection (ATCC, Manassas, VA, USA). All the cells were grown in Dulbecco's Modified Eagle's Medium (DMEM) containing 10% fetal bovine serum (FBS; Gibco/Thermo Fisher Scientific, Grand Island, NY, USA) and 100 units/mL penicillin and streptomycin (Gibco) in a humidified incubator at 37°C and 5% CO_2_.

### Preparation of exosomes

Exosomes were separated from the serum and the cell culture medium by first filtering through a 0.45–lm PVDF filter (Millipore, Billerica, MA) to eliminate cellular debris. ExoQuick Exosome Precipitation Solution (SBI System Biosciences, Mountain View, California, USA) was added to the supernatant and mixed well. The mixture was precipitated by incubating at –4°C for 30 min. The exosomes were pelleted by centrifuging the mixture at 1,500 × g for 30 min at 4^°^C. The exosome pellets were re-suspended in PBS and stored at –80°C for further experiments.

### Transmission electron microscopy

The exosomes were diluted to 1 mg/ml in PBS and spotted on a glow-discharged copper grid on the filter paper, which was subsequently dried for 20 min using the infrared lamp. The grid was stained with 2% uranyl acetate (pH 7.0) for 40 seconds and then air-dried at room temperature. Exosomes were examined under transmission electron microscopy (H-600 HITACHI microscope, Japan) operated at 80 kV.

### Western blotting

The exosomal proteins were extracted in 10× protein lysis buffer and centrifuged twice at 20,000 × *g* for 15 min at 4^°^C. The protein supernatant was quantified by the bicinchoninic acid assay (BCA) method. Equal amounts of proteins were separated by sodium dodecyl sulfate-polyacrylamide gel electrophoresis (SDS-PAGE). Then, the separated proteins were transferred onto a PVDF membrane (Bio-Rad, Hercules, CA, USA). After blocking the membrane with 5% skimmed milk, the blots were incubated with anti-CD63 antibody (1:3000, Ab84618; Abcam, Cambridge, UK), anti-TSG101 antibody (1:2000, Ab12011; Abcam), and anti-Hsp70 antibody (1:1000, Ab45133; Abcam) against the exosomal specific CD63, TSG101 and Hsp70 protein overnight at 4°C. Then, after washing, the blots were incubated with the HRP-conjugated secondary anti-Rabbit antibody at room temperature for 1 h. After washing, the blots were developed with enhanced chemiluminescent detection kit (Amersham, London, UK), exposed to X-ray film, and the protein bands were quantified by densitometry using a video documentation system (Gel Doc 2000, Bio-Rad).

### RNA isolation and detection of exosomal CRNDE-p and miR-217 by qRT-PCR

Exosomal RNA was processed by MirVana microRNA isolation kit (Life Technologies, Carlsbad, Calif). Then, the exosomal CRNDE-p and miR-217 expression was determined by qRT-PCR using NCODE CE SYBR GREEN MIRNA kit (Life Technologies, Carlsbad, Calif) according to manufacturer's instructions. During RNA isolation, 2 μL synthetic Caenorhabditis elegans cel-miR-39 (RiboBio, Guangzhou, China) was added as a spike-in control in all samples. For cDNA synthesis, 1 μg total RNA from each serum exosomal sample was incubated with the following mixture: 5 × Reaction Mix, 4 μl; 10 ×SuperScript Enzyme Mix, 2 ml and DEPC treated water to 20 μl in the reaction tube. The mixture was incubated at 37°C for 60 min and terminated at 95°C for 5 min. Quantitative PCR analysis was performed in the ABI PRISM 7900 Sequence Detector System (Applied Biosystems, Foster City, CA, USA). The qPCR primers for CRNDE-p, miR-217 and cel-miR-39 were purchased from RiboBio. The relative expression levels of CRNDE-p and miR-217 were normalized against cel-miR-39 by the comparative 2^−ΔΔCt^ method. All experiments were performed in triplicates.

### CRC mouse xenograft model

1 × 10^7^ HCT-116 cells were suspended in a solution of 50% basement membrane matrix (BD Matrigel) in PBS and injected subcutaneously into six male BALB/c nude mice (6-week-old). Another six mice were mock injected with 200μl ice-cold 50% Matrigel in PBS. After 5 weeks, their blood was collected in a procoagulant tube by eyeball enucleation and the mice were euthanized. The exosomal preparation and RNA isolation were performed similar to human serum samples.

### Statistical analysis

Statistical analysis was performed using SPSS19.0 statistical software. Data between two groups was compared by student's *t* test. Multiple comparisons of measurement data were performed by one-way ANOVA. Pairwise comparisons of multiple averages were performed by the Kruskal–Wallis test. Receiver-operating characteristic (ROC) curves and the area under the ROC curve (AUC) were used to calculate sensitivity and specificity and thereby assess the diagnostic accuracy.
